# Supplementary material for: Coagulation Factor XIIIa and Activated Protein C Activate Platelets via GPVI and PAR1
Source: Int J Mol Sci. 2022 Sep 6;23(18):10203. doi: 10.3390/ijms231810203 (PMC9499330; doi:10.3390/ijms231810203)
Supplement: Supplementary file 1 [file ijms-23-10203-s001.zip › ijms-1888257-supplementary.pdf]

Supplementary materials

Coagulation factor XIIIa and activated protein C activate platelets via GPVI and PAR1

Ilaria De Simone [1,2], Constance C. F. M. J. Baaten [1,3], Martine Jandrot-Perrus [4], Jonathan M. Gibbins [2], Hugo ten Cate [1,5], Johan W. M. Heemskerk [1,6], Chris I. Jones [2,*], Paola E. J. van der Meijden [1,5,*]

1 Department of Biochemistry, Cardiovascular Research Institute Maastricht (CARIM), Maastricht University, 6200 MD Maastricht, The Netherlands
2 School of Biological Sciences, Institute for Metabolic and Cardiovascular Research, University of Reading, RG6 6AS Reading, UK
3 Institute for Molecular Cardiovascular Research, University Hospital Aachen, RWTH Aachen University, 52074 Aachen, Germany
4 UMR_S1148, Laboratory for Vascular Translational Science, INSERM, University Paris Cité, F-75018 Paris, France
5 Thrombosis Expertise Center, Heart and Vascular Center, Maastricht University Medical Center, 6229 HX, Maastricht, The Netherlands
6 Synapse Research Institute, 6217 KD Maastricht, The Netherlands
* equal contribution
* Correspondence:
Chris I. Jones, School of Biological Sciences, Institute for Cardiovascular and Metabolic Research, University of Reading, RG6 6AS Reading, UK. Tel: +44-(0)-118-378-7047. E-mail: c.i.jones@reading.ac.uk

Paola E. J. van der Meijden, Department of Biochemistry Maastricht University P.O. Box 616, 6200 MD, Maastricht, The Netherlands Tel.: +31 43 3881684, E-mail: p.vandermeijden@maastrichtuniversity.nl

Materials

Collagen-related peptide cross-linked (CRP-XL) was purchased from Prof. Richard Farndale (University of Cambridge, UK). Human α−thrombin came from Kordia (Leiden, The Netherlands. PAR1-activating peptide TRAP6 (SFLLRN) was from Bio Connect (Toronto, Canada); ADP and honokiol were from Sigma (Zwijndrecht, The Netherlands). Collagen-I Horm derived from equine tendon was from Nycomed (Hoofddorp, The Netherlands). Von Willebrandt factor (VWF) was from Invitrogen (Waltham, Massachusetts, United states). Human FXa was from ERL (Enzyme Research Laboratories, Swansea, UK). Human FXIIIa (A subunit, recombinantly produced in insect cells) and the transglutaminase inhibitor T101 were from Zedira (Darmstadt, Germany). The human recombinant activated protein C (APC) was produced, as described elsewhere [1]. Dabigatran etexilate was purchased from Alsachim (Illkirch Graffenstaden, France). Atopaxar was purchased from Axon Medchem (Groningen, the Netherlands). The selective spleen tyrosine kinase (Syk) inhibitor PRT 060318, 2-((1R,2S)-2-aminocyclohexylamino)-4-(m-tolylamino)pyrimidine-5-carboxamide (Syk-IN), came from Bio-Connect (Huissen, the Netherlands). Tirofiban (aggrastat) from Merck Sharp & Dohme. Anti-GPVI Fab 9O12 was a kind gift from Dr. M. Jandrot-Perrus (INSERM, University Paris Diderot, Paris, F) [2]. (FITC)-conjugated PAC-1 antibody against active integrin $\alpha_{IIb}\beta_3$ was from Becton-Dickinson Bioscience (Franklin Lakes, NJ, USA). Alexa Fluor (AF)647-labelled anti-human CD62P mAb was from Biolegend (San Diego, California, United States).

Preparation of washed platelets

Blood was first centrifuged for 15 min at 258 g (22°C, acc. 9, brake 0; Rotina 380R, Hettich Benelux B.V., Geldermalsen, The Netherlands) to obtain platelet-rich plasma (PRP). PRP was collected and 1:10 Acid Citrate Dextrose (ACD; 80 mM Tri-sodium citrate (.2H2O), 52 mM Citric acid (.H2O), 183 mM D-(+)-glucose) was added, whereafter PRP was centrifuged at 2200 g for 2 min (22°C; Hettich EBA 12, Hettich Benelux B.V., Geldermalsen, The Netherlands). The platelet pellet was resuspended in Hepes buffer pH 6.6 (10 mM Hepes, 136 mM NaCl, 2.7 mM KCl, 2 mM $MgCl_2$, 0.1% glucose and 0.1 % BSA), whereafter Apyrase (0.1 U/ml) and ACD (1:15) were added. After another centrifugation step (2200 g, 2 min, 22°C; Hettich EBA 12, Hettich Benelux B.V., Geldermalsen, The Netherlands), platelets were resuspended in Hepes buffer pH 7.45 (10 mM Hepes, 136 mM NaCl, 2.7 mM KCl, 2 mM $MgCl_2$, 0.1% glucose and 0.1 % BSA) as described earlier [3].

Cytosolic $[Ca^{2+}]_i$ measurements

Washed human platelets (200×10^9/L) were incubated with Fura-2 acetoxymethyl ester (3 μM) and pluronic (0.4 μg/ml) for 40 minutes at room temperature. After washing away the residual probe, the Fura-2 loaded platelets were resuspended in Hepes buffer pH 7.45 and changes in cytosolic $[Ca^{2+}]_i$ were measured in 96-well plates using a FlexStation 3 (Molecular Devices, San Jose, CA, USA). In brief, after adding 1 mM $CaCl_2$, the platelets were stimulated by automated pipetting 10 μg/ml FXa. Changes in Fura-2 fluorescence were continuously measured over time at 37 °C by ratiometric fluorometry at excitation wavelengths of 340 and 380 nm, and an emission wavelength of 510 nm. After correction for background fluorescence, ratio values were used to calculate $[Ca^{2+}]_i$ (nM).

Flow cytometric analysis

Integrin $\alpha_{IIb}\beta_3$ activation was measured via FITC-conjugated PAC-1 chimeric Ab (1.25 μg/ml), which binds the active integrins. Platelet granular secretion was measured by P-selectin expression, detected using Alexa Fluor (AF)647-labelled antihuman CD62P mAb (2.5 μg/ml).

For the experiment assessing the effect of SCP on platelet activation, washed platelets (500x10^9 platelets/L) were resuspended in SCP or Hepes buffer (pH 7.45) at a 1:5 ratio and were then stimulated with a range of CRP-XL concentrations (0.003-3 μg/ml) for 10 minutes, in the presence of 2 mM $CaCl_2$. Thereafter, platelets were fixed for at least 10 minutes, by addition of formyl saline (0.2% formaldehyde in 0.15 M NaCl).

For all other flow cytometry experiments, washed platelets (50x10^9 platelets/L) were stimulated with a range of CRP-XL (0.03-0.5 μg/mL) concentrations in the presence of PAC-1 to determine a suboptimal agonist concentration per donor (i.e. the agonist concentration generating 40-60% PAC-1 positive platelets). Platelets were then stimulated with a suboptimal agonist concentration combined with FXIIIa (10 U/ml), APC (10 nM) and/or FXa (10 μg/ml), in the presence of 2mM $CaCl_2$. Coagulation factor concentrations were as used previously,[4-6] presumed that physiologically at least 1/10 of the plasma zymogen concentration is activated. Platelets were stimulated for 10 minutes, whereafter they were fixed for at least 10 minutes, by addition of formyl saline (0.2% formaldehyde in 0.15 M NaCl). 5000 platelets per sample were measured using a BD Accuri C6 flow cytometer and analysed with CFlow Plus software (BD Bioscience, Franklin Lakes, New Jersey, United States).

Plate-based aggregation

Measurements of aggregation were performed using 96-well plates (Greiner) containing the following platelet agonists at a range of concentrations: ADP, CRP-XL, TRAP6, Thrombin. No additional fibrinogen was added. Washed platelets resuspended in SCP and control plasma, or washed platelets in Hepes buffer (400x10^9 platelets/L) isolated from the citrated blood of healthy donors was loaded onto

plates and shaken at 1200 rpm for 5 minutes at 37°C using a plate shaker (Quantifoil Instruments), as described earlier;[7] absorption of 405 nm light was measured using a FlexStation 3 (Molecular Devices, San Jose, CA, USA).

Platelet spreading

Washed glass coverslips were coated with coagulation factors at concentrations as used previously [4-6], FXIIIa (10 U/ml), recombinant APC (10 nM) and blocked with 1% BSA. Washed platelets ($20x10^9$ platelets/L) were allowed to adhere and spread for 45 minutes at 37°C. Non-adherent platelets were removed by gently washing the slides three times with Hepes buffer, pH 7.45. Then, remaining adherent platelets were fixed for 10 minutes, using 1% paraformaldehyde solution. After washing, fixated platelets were permeabilized with 0.005% SDS in phosphate-buffered saline (PBS) for 10 minutes and subsequently blocked with 5% BSA in PBS during 20 minutes. F-actin was stained using phalloidin (1U/mL CF543-phalloidin in 1% BSA in PBS) for 1 hour at room temperature, after which fluorescence images were captured using an EVOS-FL microscope (Life Technologies, Bleiswijk, the Netherlands), equipped with Cy5, RFP, and GFP LEDs; an Olympus UPLSAPO 60× oil immersion objective; and a sensitive 1360×1024 pixel CCD camera. The platelets were scored as being adherent, extending filopodia and fully spread (formation of lamellipodia).

Whole blood perfusion assays

Platelet adhesion and aggregation under flow was studied using the Maastricht parallel-plate flow chamber [8]. Washed coverslips were coated with 0.5 µl microspots, containing VWF (12.5 µg/ml) with or without FXIIIa (200 µg/ml) or recombinant APC (20 nM). The coated coverslips were incubated in a humid chamber for 1 hour, then washed with saline and blocked for 30 minutes with blocking buffer (1% BSA in HEPES buffer, pH 7.45). When indicated, citrated blood was incubated with FXIIIa (10 U/ml), while blood taken on hirudin was incubated with APC (10 nM), for 5 minutes. Subsequently, the citrated blood was recalcified with 3.75 mM $MgCl_2$ and 7.5 mM $CaCl_2$ in the presence of 40 µM PPACK, whereafter it was perfused over a coverslip for 3.5 min or 5 min at a wall-shear rate of 1000 s^{-1} or $300s^{-1}$, respectively. Brightfield images were taken per microspot, using the EVOS-FL microscope (Life Technologies, Bleiswijk, the Netherlands); an Olympus UPLSAPO 60× oil immersion objective; and a sensitive 1360 × 1024 pixel CCD camera. Image analysis was performed using the program Fiji [9]. Parameters extracted from brightfield images were 'platelet surface area coverage' (% PltSac), which is the percentage of area covered with adhered platelets, and 'Microaggregates' (% MicrAgg), which is the percentage of area covered by microaggregates.

Supplementary Figure

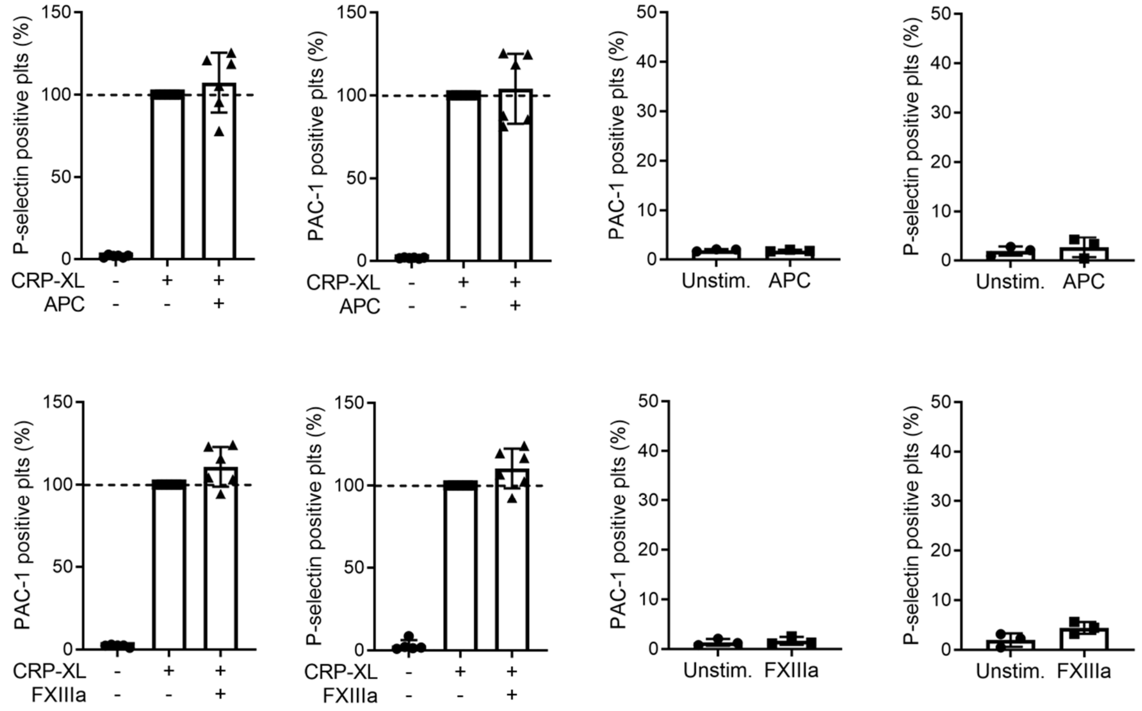

Supplementary Figure S1. Individual, soluble (anti-)coagulation factors APC and FXIIIa do not affect CRP-XL induced platelet activation. Washed platelets were preincubated with vehicle or FXIIIa and APC and activated with a submaximal CRP-XL concentration (0.03-0.5 µg/mL). Data scaled relative to CRP-XL. Flow cytometry was used to measure activated integrin $\alpha_{IIb}\beta_3$ using FITC labelled PAC-1 mAb and P-selectin expression using Alexa Fluor (AF)647-labelled antihuman CD62P mAb. One sample t-test with false discovery rate correction, mean ± SD, *n* = 4.

References

1. Wildhagen KC, Schrijver R, Beckers L, ten Cate H, Reutelingsperger CP, Lutgens E, Nicolaes GA. Effects of exogenous recombinant APC in mouse models of ischemia reperfusion injury and of atherosclerosis. PLoS One. 2014;9(7):e101446. Epub 2014/07/18. doi:10.1371/journal.pone.0101446. Cited in: Pubmed; PMID 25032959.

2. Lecut C, Feeney LA, Kingsbury G, Hopkins J, Lanza F, Gachet C, Villeval JL, Jandrot-Perrus M. Human platelet glycoprotein VI function is antagonized by monoclonal antibody-derived Fab fragments. J Thromb Haemost. 2003 Dec;1(12):2653-62. Epub 2003/12/17. doi:10.1111/j.1538-7836.2003.00495.x. Cited in: Pubmed; PMID 14675102.

3. Gilio K, Munnix IC, Mangin P, Cosemans JM, Feijge MA, van der Meijden PE, Olieslagers S, Chrzanowska-Wodnicka MB, Lillian R, Schoenwaelder S, Koyasu S, Sage SO, Jackson SP, Heemskerk JW. Non-redundant roles of phosphoinositide 3-kinase isoforms alpha and beta in glycoprotein VI-induced platelet signaling and thrombus formation. J Biol Chem. 2009 Dec 4;284(49):33750-62. Epub 2009/10/10. doi:10.1074/jbc.M109.048439. Cited in: Pubmed; PMID 19815551.

4. White TC, Berny MA, Tucker EI, Urbanus RT, de Groot PG, Fernandez JA, Griffin JH, Gruber A, McCarty OJ. Protein C supports platelet binding and activation under flow: role of glycoprotein Ib and apolipoprotein E receptor 2. J Thromb Haemost. 2008 Jun;6(6):995-1002. Epub 2008/05/21. doi:10.1111/j.1538-7836.2008.02979.x. Cited in: Pubmed; PMID 18489431.

5. Magwenzi SG, Ajjan RA, Standeven KF, Parapia LA, Naseem KM. Factor XIII supports platelet activation and enhances thrombus formation by matrix proteins under flow conditions. J Thromb Haemost. 2011 Apr;9(4):820-33. Epub 2011/02/16. doi:10.1111/j.1538-7836.2011.04234.x. Cited in: Pubmed; PMID 21320283.

6. Petzold T, Thienel M, Dannenberg L, Mourikis P, Helten C, Ayhan A, M'Pembele R, Achilles A, Trojovky K, Konsek D, Zhang Z, Regenauer R, Pircher J, Ehrlich A, Lusebrink E, Nicolai L, Stocker TJ, Brandl R, Roschenthaler F, Strecker J, Saleh I, Spannagl M, Mayr CH, Schiller HB, Jung C, Gerdes N, Hoffmann T, Levkau B, Hohlfeld T, Zeus T, Schulz C, Kelm M, Polzin A. Rivaroxaban Reduces Arterial Thrombosis by Inhibition of FXa-Driven Platelet Activation via Protease Activated Receptor-1. Circ Res. 2020 Feb 14;126(4):486-500. Epub 2019/12/21. doi:10.1161/CIRCRESAHA.119.315099. Cited in: Pubmed; PMID 31859592.

7. Swieringa F, Spronk HMH, Heemskerk JWM, van der Meijden PEJ. Integrating platelet and coagulation activation in fibrin clot formation. Res Pract Thromb Haemost. 2018 Jul;2(3):450-460. Epub 2018/07/27. doi:10.1002/rth2.12107. Cited in: Pubmed; PMID 30046749.

8. de Witt SM, Swieringa F, Cavill R, Lamers MM, van Kruchten R, Mastenbroek T, Baaten C, Coort S, Pugh N, Schulz A, Scharrer I, Jurk K, Zieger B, Clemetson KJ, Farndale RW, Heemskerk JW, Cosemans JM. Identification of platelet function defects by multi-parameter assessment of thrombus formation. Nat Commun. 2014 Jul 16;5:4257. Epub 2014/07/17. doi:10.1038/ncomms5257. Cited in: Pubmed; PMID 25027852.

9. Schindelin J, Arganda-Carreras I, Frise E, Kaynig V, Longair M, Pietzsch T, Preibisch S, Rueden C, Saalfeld S, Schmid B, Tinevez JY, White DJ, Hartenstein V, Eliceiri K, Tomancak P, Cardona A. Fiji: an open-source platform for biological-image analysis. Nat Methods. 2012 Jun 28;9(7):676-82. Epub 2012/06/30. doi:10.1038/nmeth.2019. Cited in: Pubmed; PMID 22743772.
